# Analyzing Alkyl Bromide Genotoxic Impurities in Febuxostat Based on Static Headspace Sampling and GC-ECD

**DOI:** 10.3390/ph17040422

**Published:** 2024-03-26

**Authors:** Alexandros Kavrentzos, Elli Vastardi, Evangelos Karavas, Paraskevas D. Tzanavaras, Constantinos K. Zacharis

**Affiliations:** 1 Laboratory of Pharmaceutical Analysis, Department of Pharmacy, Aristotle University of Thessaloniki, 54124 Thessaloniki, Greece; akavrentzos@pharmathen.com; 2 Pharmathen S.A. Pharmaceutical Industry, Dervenakion Str 6. Pallini Attikis, 15351 Athens, Greece; evastardi@pharmathen.com (E.V.); ekaravas@pharmathen.com (E.K.); 3 Laboratory of Analytical Chemistry, Department of Chemistry, Aristotle University of Thessaloniki, 54124 Thessaloniki, Greece; ptzanava@chem.auth.gr

**Keywords:** genotoxic impurity, headspace sampling, gas chromatography, febuxostat, experimental design

## Abstract

Herein, a sensitive and selective gas chromatography-electron capture detector (GC-ECD) method was developed and validated for the quantification of trace levels of five bromo-containing genotoxic impurities in Febuxostat active pharmaceutical ingredient (API) after headspace sampling (HS). Multivariate experimental designs for the optimization of static headspace parameters were conducted in two stages using fractional factorial design (FFD) and central composite design (CCD). The optimum headspace conditions were 5 min of extraction time and a 120 °C extraction temperature. Baseline separation on the analytes against halogenated solvents was carried out using an Agilent DB-624 (30 m × 0.32 mm I.D., 1.8 μm film thickness) stationary phase under isothermal conditions. The method was validated according to ICH guidelines in terms of specificity, linearity, the limits of detection and quantification, precision and accuracy. The linearity was assessed in the range of 5–150% with respect to the specification limit. The achieved LOD and LOQ values ranged between 0.003 and 0.009 and 0.01 and 0.03 μg mL^−1^, respectively. The accuracy of the method (expressed as relative recovery) was in the range of 81.5–118.2%, while the precision (repeatability, inter-day) was less than 9.9% in all cases. The validated analytical protocol has been successfully applied to the determination of the impurities in various Febuxostat API batch samples.

## 1. Introduction

Febuxostat, known chemically as 2-(3-cyano-4-isobutoxyphenyl)-4-methyl-1,3-thiazole-5-carboxylic acid, is a xanthine oxidase inhibitor, and it is used for the treatment of hyperuricemia in patients with chronic gout [1]. Several studies have exhibited that febuxostat reduces inflammatory responses by lowering the levels of pro-inflammatory mediators [2]. It shows higher efficacy and tolerability compared to allopurinol and is often prescribed for patients who are unable to take allopurinol [3]. It was approved by the FDA in 2009 and is branded at dosage forms of 40 and 80 mg/tab.

Genotoxic impurities may originate from different sources during the synthesis of an API, and they are mainly introduced as starting materials, by-products or intermediates [4]. In the case of febuxostat, many reagents, starting materials and intermediates are utilized, including *n*-propyl bromide (nPrBr), isopropyl bromide (isoPrBr), *n*-butyl bromide (nBuBr), isobutyl bromide (isoBuBr) and sec-butyl bromide (secBuBr) [5]. These compounds are generally toxic and cancer suspect agents. The European Agency for the Evaluation of Medicinal Products (EMEA) and the FDA have determined the acceptable intake of a compound that does not present a significant risk of carcinogenicity or toxicological effects [6]. Based on the threshold of toxicological concern (TTC) and the maximum daily dosage of febuxostat, the above potential genotoxic impurities (PGIs) must be less than 13 ppm (μg g^−1^) as the cut-off level in the API plant batch samples. 

The development and validation of sensitive analytical methods for the determination of PGIs are of great importance to the pharmaceutical industry. Depending on the volatility of the analytes, these analytical schemes are mainly based on the usage of liquid or gas chromatography coupled with highly sensitive detectors such as mass spectrometers (MS), electron capture detectors (ECD), fluorescence (FLD), etc. Looking at the determination of volatile organic impurities (OVIs), GC is considered the “gold standard” due to its high separation efficiency and sensitivity. Such analysis can be performed by either direct injection or HS sampling, with the latter being categorized in dynamic and static HS [7]. Static HS is more straightforward than the dynamic one, and it has been widely utilized in the analysis of OVIs in drug substances and products [7,8,9,10]. This technique is approved by ICH and various pharmacopeias. Its principle is based on the thermostatic partitioning of volatile compounds between the diluent (solvent with a high boiling point) and the gas phase. Compared to the direct injection, the HS approach reduces the number of non-volatile matrix components that can be injected into the GC instrument by sampling only the gaseous component in a sealed sample vial. Consequently, the matrix effect is minimized, resulting in high method sensitivity [11]. Commonly used sample diluents for HS GC analysis include dimethylsulfoxide (DMSO), *N*,*N*-di-methylformamide, *N*,*N*-dimethylacetamide, benzyl alcohol, water, their mixtures and even ionic liquid [12,13,14]. 

Optimizing the HS conditions is crucial for achieving both high method sensitivity and reproducibility. This procedure can be carried out through either univariate or multivariate approaches. The first procedure does not take into consideration the possible interactions between factors and may provide misleading results. On the contrary, the multivariate approach (DoE) allows for the maximum utilization of data from a set of experiments providing a holistic understanding of the significance of the factors and their interaction [15]. Utilizing the mathematical criteria embedded in factorial design, this approach facilitates the prediction of optimal values for the experimental parameters. 

To the best of our knowledge, no analytical method for the quantitation of the five bromo-containing genotoxic impurities in febuxostat API has been reported. Another publication in the literature reports the determination of the methyl bromide, ethyl bromide, isoProBr, nPrBr and nBuBr in divalproex sodium using GC-MS [16]. In this study, we report a systematic approach for the analysis of the aforementioned PGIs (nPrBr, isoPrBr, nBuBr, isoBuBr and secBuBr) using GC-ECD after HS sampling. The DoE approach was utilized for the optimization of the HS experimental conditions. The developed analytical scheme was validated according to ICH guidelines and finally applied to the various Febuxostat API batches.

## 2. Results and Discussion

### 2.1. Optimization of Separation Conditions

The development of a GC-ECD method for the separation and analysis of certain PGIs is vital for a fast and reliable turnaround of analytical results. Since the API samples may contain other OVIs, i.e., methanol (MeOH), ethanol (EtOH), acetonitrile (ACN), benzene (BNZ), tetrahydrofuran (THF), ethyl acetate (EtOAc), dichloromethane (DCM), dimethylformamide (DMF) and acetic acid (AcA), a mixture of these solvents with the analytes was utilized for the study and optimization of the separation conditions. As expected, only DCM was detected using ECD, which was successfully separated from isoPrBr at low oven temperatures (i.e., 45 °C) using the DB-624 as a stationary phase. However, no baseline separation between secBuBr and isoBuBr was obtained at these conditions. Taking into account that both compounds have almost identical boiling points (ca 91 °C), experimental trials were conducted using lower oven temperatures. Finally, an oven temperature of 35 °C was chosen for the first 25 min of the analysis, resulting in a resolution of 1.6 between the respective impurities. After that period, a ramp of 25 °C/min followed by a hold time of 2 min at 240 °C was implemented for column cleanup, avoiding potential carry-over effects. Higher ramp values were not tested to prevent obtaining a noisy and upward slope baseline.

The split ratio was investigated in a range of 1:10–1:50 to enhance the sensitivity of the PGIs analysis. Theoretically, a reduction in the inlet split ratio results in the injection of higher sample amounts and, therefore, an improvement in the method sensitivity. As anticipated, a lower split ratio led to a significant improvement in the sensitivity of all tested compounds, and therefore, the value of 1:10 was adopted for subsequent experiments.

### 2.2. Study of HS Conditions

During the HS sampling, several critical parameters such as the incubation temperature and time, agitation speed, sample volume, etc. should be studied and optimized. The univariate approach (one-factor-at-a-time, OFAT) for optimization does not consider the possible interactions between the variables, leading to misleading results. On the other hand, the chemometric approach provides global knowledge by revealing significant factors and interactions [15]. The DMSO was selected as the diluent throughout this study due to its excellent solubility of the API samples and the minimal interference with the earlier eluting compounds in the GC assay.

#### 2.2.1. Screening of HS Parameters

**Figure 1:**
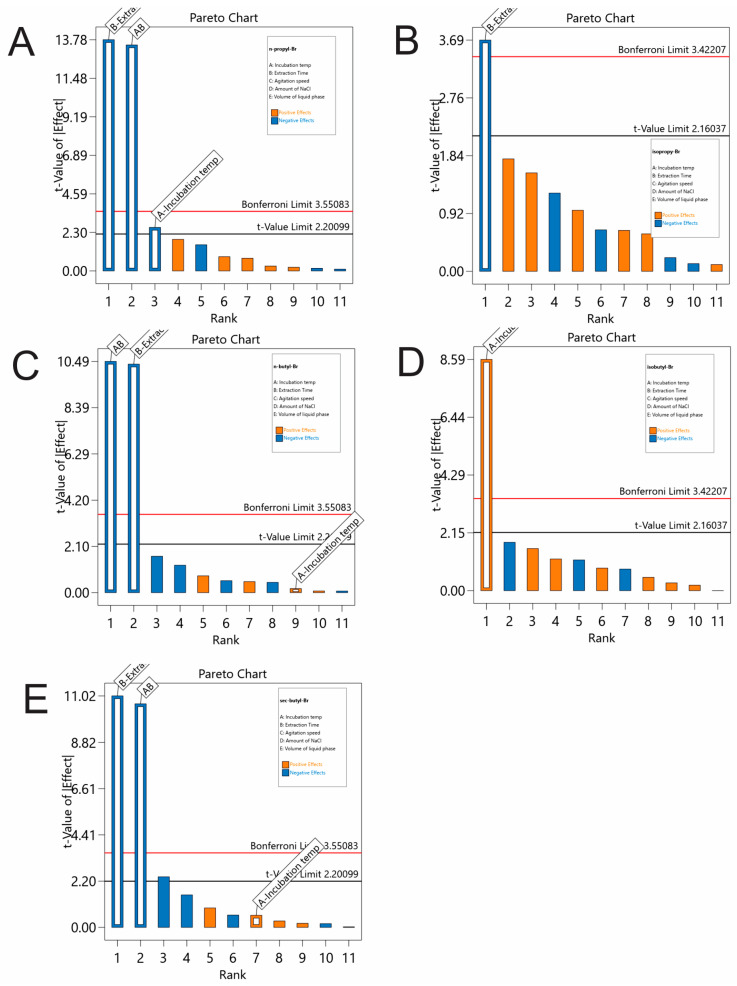
Pareto charts (sorted by order of importance) of the studied parameters for the peak area of (**A**) nPrBr, (**B**) isoPrBr, (**C**) nBuBr, (**D**) isoBuBr and (**E**) secBuBr.

Among other HS experimental parameters affecting the efficiency of the HS, two instrumental (incubation temperature and stirring rate) and three sample preparation variables (incubation time, salt amount concentration and sample volume) were initially screened. The ranges of each parameter were the following: incubation temperature (80–120 °C, Factor A), extraction time (5–30 min, Factor B), agitation speed (250–750 rpm, Factor C), NaCl amount concentration (0–10% *w*/*v*, Factor D) and sample volume (1–5 mL, Factor E). For this purpose, a minimum-run screening design (resolution IV) was built, consisting of 12 factorial and 3 central points, using the Design-Expert 13 software (vs 22.0.8, Stat-Ease^®^ Inc., Minneapolis, MN, USA). The experiments were run randomly to minimize the effect of uncontrolled variables. The significance of the analytical condition is assessed through the *p*-value (<0.05) obtained by the ANOVA of multivariate regression analysis (95% confidence interval). The examined variables and their ranges are reported in Table S1 (Supplementary Materials). A graphical analysis of the Pareto charts (Figure 1) revealed that the incubation temperature and the extraction time have statistically significant positive effects on the peak areas of isoBubr, while the extraction time was significant on the rest of the responses.

The interaction A × B also had a significant effect on the peak areas of nPrBr, nBuBr and secBuBr. The rest of the variables (agitation speed, NaCl amount concentration and sample volume) were non-significant in the examined ranges, as their main effects were less than the Bonferroni and t-value limits. To summarize, the extraction time and incubation temperature were further examined by RSM, and the other values were set as follows: agitation speed, 250 rpm; NaCl amount concentration, 0% *m*/*v*; sample volume, 3 mL.

#### 2.2.2. Optimization of HS Parameters

The subsequent step involved the optimization of the two HS parameters through the utilization of central composite design (CCD) [17]. It was constructed by fractionating the experimental range for each factor into three levels (face-centered), with the star points positioned at the center of each face of the factorial design, maintaining the design resolution (Type V). The same experimental domain with the screening design was investigated. A set of 13 runs were performed, including 6 points at the center of the experimental domain, to estimate the experimental error required for the assessment of the lack of fit of the model [17]. Table S2 tabulates the factorial design points and their measured responses, where the runs were randomized to avoid systematic errors. Through the application of multivariate regression analysis, a fitted linear or quadratic model was developed to obtain a predictive model for each response. High-order models such as cubic models were found to be aliased for all responses, suggesting the augmentation of the design.

**Figure 2:**
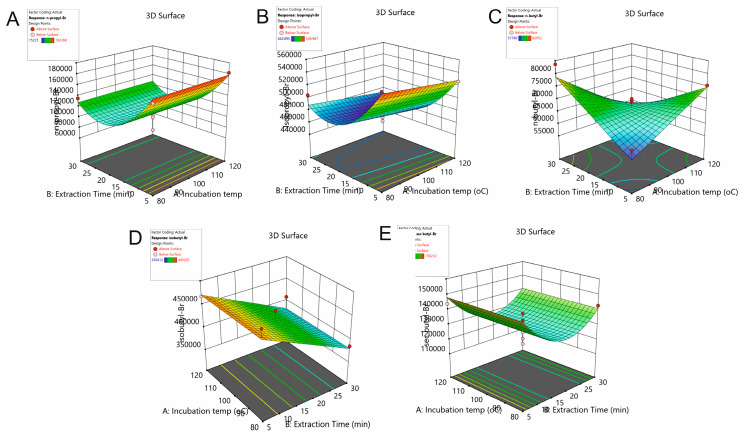
3D response surface plots showing the effects of the incubation temperature and extraction time on the peak area of (**A**) nPrBr, (**B**) isoPrBr, (**C**) nBuBr, (**D**) isoBuBr and (**E**) secBuBr.

Table 1 shows the acquired regression models along with the relevant statistical parameters derived from ANOVA tests. The non-significant factors (*p* > 0.05) have been omitted from the models using the backward elimination approach. The peak areas of nBuBr and isoBuBr follow a simple linear relationship with the studied variables, and the rest of the responses are described by a second-order quadratic model. The response surface of the studied responses is shown in Figure 2. All models were found to be significant, and the *R*^2^ values were >0.7048, implying goodness of fit and adequate predictability. The adequate precision was >8.0, indicating the significance of the models.

The “lack of fit” (LoF) was found to be non-significant relative to the pure error. The validity of the models is assessed by examining the normal probability plot of residuals and the plot of the residuals compared to the predicted values. A random scatter of the experimental data around the line was observed, revealing proper model fitting (Figures S1 and S2, Supplementary Materials). The ANOVA results are given in Tables S3–S7.

**Figure 3:**
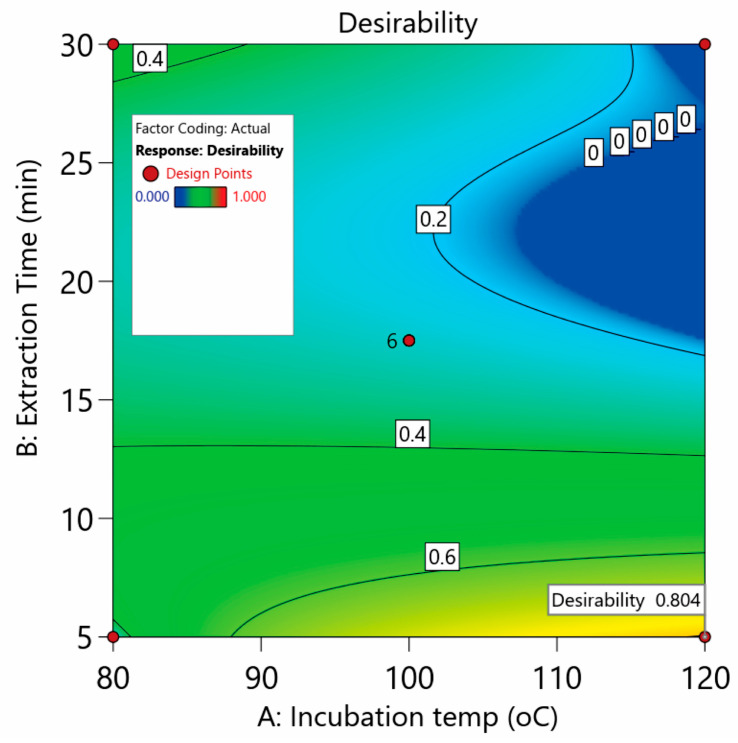
Desirability counter plot for defining the optimal conditions of the studied responses.

Undoubtedly, an improved overall extraction of the studied compounds is the main advantage of our method. On this basis, Derringer’s desirability function was utilized as the geometrical mean of the five individual desirability values *d*_i_ (weight factor = 1) for each response. The numerical optimization led to a global desirability of 0.804 (Figure 3). The optimum values were found to be 120 °C and 5 min for the incubation temperature and extraction time, respectively.

#### 2.2.3. Robustness of HS Conditions Using Monte-Carlo Simulations

**Figure 4:**
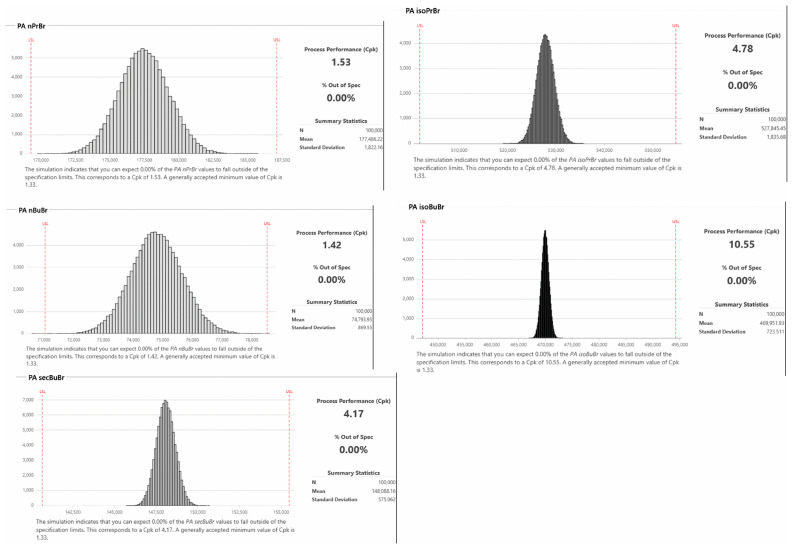
Probabilistic distribution of the peak area of the analytes during Monte-Carlo simulation experiments.

The robustness of the HS method was investigated using Monte-Carlo simulations and capability analysis. For this purpose, 100 *k* iterations were performed using Monte-Carlo simulation experiments, and the simulated data were utilized to estimate the Cpk values. The acceptance criteria of the peak area of analytes were established to ± 5% of the predicted value obtained from the optimization step. First, a group of simulation experiments was performed considering the mean value of 120 °C (temperature) and 5 min (extraction time), with standard deviation (SD) values of 1 and 0.5, respectively. The analysis revealed that the CpK values for all analytes were >1.33, except for nPrBr, showing that 5.22% of the results will be out of specification, suggesting minimization of the SD values. The readjustment of the SD value of the most robustness-sensitive parameter (extraction time) resulted in satisfactory CpK values of 1.49, 4.77, 1.43, 10.26 and 4.21 at SD values of 2 and 0.2 for the incubation time and extraction, respectively. Figure 4 illustrates the histogram from the capability analysis of the examined responses.

### 2.3. Method Validation

The developed method was validated by assessing the specificity, linearity, repeatability, intermediate precision, accuracy, LOD and LOQ according to ICH guidelines [18].

**Figure 5:**
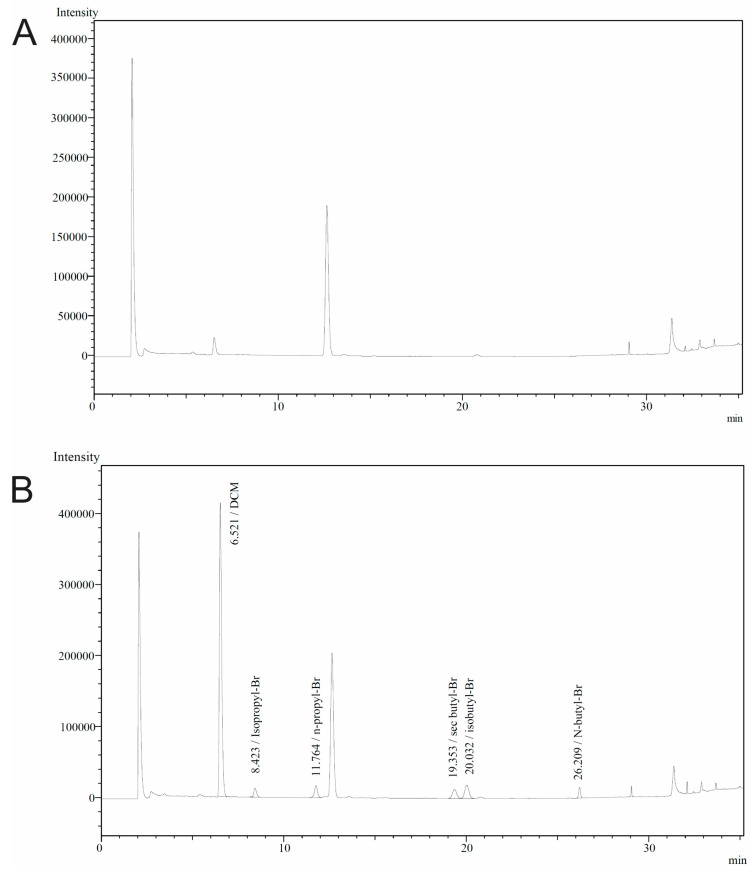
Representative HS-GC-ECD chromatograms of (**A**) the blank Febuxostat sample and (**B**) the sample spiked with PGIs (1.3 μg mL^−1^) and MeOH (300 μg mL^−1^), EtOH (500 μg mL^−1^), ACN (41 μg mL^−1^), DCM (60 μg mL^−1^), EtOAc (500 μg mL^−1^), THF (72 μg mL^−1^), BNZ (0.2 μg mL^−1^) and DMF (88 μg mL^−1^).

The specificity was investigated by analyzing a blank API sample and spiked with the respective PGIs and other OVIs at the specification limit, as per general ICH Q3C(R8) guidelines [19]. As shown in the chromatogram of Figure 5B, the analytes were well-resolved from other impurities, indicating the method is selective for the determination of the selected impurities.

The linearity of the method was investigated in the presence and absence of Febuxostat API. Seven concentration levels ranging from 5 to 150% of the specification level (corresponding to 0.07–1.95 μg mL^−1^) were examined. For each level, three replicate extractions were made. The regression coefficients (*r*^2^) were higher than 0.9929 in all cases (Table 2). The matrix effect was assessed by calculating the CF values, which are as follows: 0.96 (nPrBr), 0.84 (isoPrBr), 1.08 (nBuBr), 0.86 (isoBuBr) and 0.90 (secBuBr).

The repeatability (within-day precision) of the proposed approach was examined at LOQ values of 50%, 100% and 150% and was prepared in triplicate. The %RSD values ranged from 5.4 to 7.3% for all analytes. Similarly, the %RSD for each analyte for three consecutive days (intermediate precision) was in the range of 4.4 to 9.9% by analyzing spiked samples on the same levels by two different analysts. An adequate method and instrument precision were observed within the analytical range of determinations.

The accuracy (expressed as relative recovery, RR %) of the method was tested by analyzing spiked Febuxostat samples at the aforementioned levels. As can be seen in Table 3, the experimental recoveries were acceptable, being in the range of 81.5–118.2%, indicating that the proposed analytical scheme has sufficient accuracy for screening and quantitating the analytes studied in the API samples.

The limit of detection (LOD) and limit of quantitation (LOQ) values were calculated based on signal-to-noise (S/N) ratios of 3 and 10, respectively. In our case, the LOD and LOQ values for all analytes ranged from 0.003 to 0.009 and from 0.01 to 0.03 μg mL^−1^, respectively. These values are adequate for this type of analysis.

The stability of the analytes in Febuxostat samples was also tested up to 36 h. Blank API samples spiked with the analytes at the specification limit were analyzed. The %RSD of the content of each PGI in the tested samples was less than 5.0%, confirming the chemical stability of the analytes in the sample matrix.

### 2.4. Sample Analysis

To demonstrate the applicability of the proposed HS-GC-ECD method, several Febuxostat API batches were analyzed. All samples were treated according to the procedure described in Section 2.2 and Section 2.3. Νo detectable levels of the examined PGIs were found, verifying the safety of the API batches. A representative chromatogram of the analysis of a Febuxostat API batch (purity 99.9%) is shown in Figure 5A.

## 3. Materials and Methods

### 3.1. Reagents and Solutions

The solvents used in this study were of purities higher than 98% and supplied from the following sources: methanol (MeOH), ethanol (EtOH), acetonitrile (ACN), benzene (BNZ), ethyl acetate (EtOAc) tetrahydrofuran (THF) (Sigma-Aldrich, Darmstadt, Germany), dichloromethane (DCM), dimethylformamide (DMF), acetic acid (AcA) and DMSO (Panreac, Barcelona, Spain). The analytes nPrBr, isoPrBr, nBuBr, secBuBr and isoBuBr was purchased from Sigma-Aldrich (Darmstadt, Germany).

Stock solutions of PGIs standard solutions were prepared in DMSO at a concentration of ca 1300 μg mL^−1^, according to GMP procedures, using volumetric flasks and glass pipettes (Grade A). Specificity solutions were prepared by dissolving 300 mg of API in 3 mL of a standard mixture containing the PGIs (1.3 μg mL^−1^ each) and OVIs, as follows: MeOH (300 μg mL^−1^), EtOH (500 μg mL^−1^), ACN (41 μg mL^−1^), DCM (60 μg mL^−1^), EtOAc (500 μg mL^−1^), THF (72 μg mL^−1^), BNZ (0.2 μg mL^−1^) and DMF (88 μg mL^−1^). Linearity standards were prepared in DMSO by serial dilutions from the stock standards solutions covering the range of ca 0.07–1.95 μg mL^−1^. Accuracy samples were prepared by dissolving 300 mg of the drug substance in a 3 mL standard mixture in DMSO at concentrations ranging from the LOQ to 150% of the specification limit.

### 3.2. GC Instrumentation and Conditions

A GC-2010 gas chromatographic system (Shimadzu, Kyoto, Japan) coupled with an electron capture detector was utilized throughout this study. HS sampling was performed using an AOC-5000 combiPal autosampler controlled using the GC-solution software (vs 2.3). The analytes were separated on an Agilent J&W DB-624 (30 m × 0.32 mm, 1.8 μm film thickness) (Part No 123–1334, SN: USD222531H) capillary column containing 6% cyanopropyl/phenyl and 94% polydimethylsiloxane stationary phase. High-purity helium gas (99.999%) was used as the carrier gas at a constant flow rate of 1.5 mL min^−1^. The injector temperature was kept at 150 °C, while the split ratio was 1:10. The oven temperature was maintained at 35 °C for the first 25 min and was then linearly increased up to 240 °C and held at 2 min. The temperature of ECD was set at 280 °C. High-purity N_2_ (99.999%) was used as a make-up gas at a flow rate of 30 mL min^−1^.

For HS sampling, a 2.5 mL glass syringe was utilized, which is thermostated at 115 °C. The sample volume was 1 mL, and the sample was incubated at 120 °C for 5 min. The sample was agitated at 250 rpm. The aspiration and the injection speed were 100 and 500 μL min^−1^, respectively.

### 3.3. Sample Preparation

About 300 mg of the Febuxostat sample was weighted and placed into a 20 mL headspace glass vial. Subsequently, 3 mL of DMSO was added, and the vial was promptly sealed with a Teflon-lined septum and a magnetic crimp cap. Then, the vial was thermostated at 120 °C for 5 min with continuous stirring to ensure the complete dissolution of the solid sample and to establish the liquid–gas equilibrium of the analytes. At a predefined time, 1 mL of the headspace was withdrawn by a glass HS syringe and injected into the GC-ECD instrument.

### 3.4. Method Validation—Calculations

The specificity of the method was studied by examining the resolution of the studied PGIs to each other and against other OVIs (namely, MeOH, EtOH, ACN, BNZ, THF, EtOAc, DCM, DMF and AcA) that were used in Febuxostat synthesis. The method is considered specific if the resolution of the analyte peak and other interferences is higher than 1.5. The linearity of the method was assessed using standard and matrix-matched standards. There were a minimum of six calibration points in the range of 5–150% of the specification limit corresponding to 0.07–1.95 μg mL^−1^. Three analyses of each level were made. Acceptable linearity was established based on a correlation coefficient *r* ≥ 0.99. The repeatability and the inter-day precision were tested at six repetitive analyses of a febuxostat sample spiked at the LOQ, at 50% and 100% of the specification level. The accuracy was evaluated at four levels: LOQ, 50%, 100% and 150% of the specification limit. Precision was considered valid when the % RSD was less than 10% and the accuracy (expressed as the % relative recovery) was in the range of 80–120%. The limit of detection (LOD) and limit of quantitation (LOQ) were estimated as the concentration having a minimum signal-to-noise ratio (*S*/*N*) = 3:1 and 10:1, respectively. Column-to-column reproducibility was investigated using two different lots of the DB-624 stationary phases. Slight modifications to the final oven temperature program can be implemented to enhance the resolution when other brands of columns are used. The stability of the standards and the spiked samples was assessed up to 36 h at ambient temperature.

For routine analysis, the quantitation of the Febuxostat samples was performed using an external standard mixture of the analytes. The experimental concentration was obtained from the following expression:

(1) $$PGI\;\left(ppm\right)\;=\;\frac{A_{sample}\times W_{STD}}{A_{STD}\times W_{sample}}\times \frac{CF}{DF}\times 10^{6}$$

where *A_sample_* and *A_STD_* are the average peak area of the respective *PGI* in the sample (duplicate analysis) and the reference standard solution (*n* = 6), respectively. *W_STD_* and *W_sample_* are the weight of the reference standard and the sample, *CF* is the correction factor (caused by the API matrix in the sample solution) and *DF* is the dilution factor.

## 4. Conclusions

A static headspace gas chromatographic method for the determination of alkyl bromide genotoxic impurities in Febuxostat API samples was developed, optimized and fully validated. DoE proved to be a viable tool for the appropriate consideration of the interactions of the instrumental and sample preparation parameters. The method presents a short sample extraction time (5 min) along with high sensitivity, with LOQ values in the range of 0.01–0.03 μg mL^−1^. Additionally, the approach is specific, accurate, linear and precise and generally meets the ICH guidelines’ requirements. It was successfully implemented in the analysis of several batches of Febuxostat API samples.

## Figures and Tables

**Table 1:** Reduced response models and statistical parameters obtained from ANOVA after backward elimination (*p* = 0.05).

Peak Area	Regression Model ^a^	F-Value (*p*-Value)	*R* ^2^	Adjusted *R*^2^	Adeq. Precision
nPrBr	2.3 × 10^5^ − 11,921 B + 283 B^2^	16.21 (0.0007)	0.7643	0.7171	8.62
isoPrBr	6.22 × 10^5^ − 389 A − 10,708 B + 242 B^2^	22.75 (0.0002)	0.8835	0.8446	14.13
nBuBr	3351 + 626.8 A + 4033.6 B − 39.9 AB	9.46 (0.0038)	0.7592	0.6790	10.74
isoBuBr	4.88 × 10^5^ − 3610 B	30.64 (0.0002)	0.7359	0.7118	14.09
secBuBr	1.65 × 10^5^ − 3898 B + 102.9 B^2^	11.94 (0.0022)	0.7048	0.6458	8.00

^a^ Significant coefficients (*p* < 0.05) are only included; A: Incubation temperature (°C), B: extraction time (min).

**Table 2:** Validation data for the quantitation of the analytes using the proposed HS-GC-ECD method.

Validation Parameter	nPrBr	isoPrBr	nBuBr	isoBuBr	secBuBr
Linear range (μg mL^−1^)	0.062–1.86	0.068–2.025	0.064–1.92	0.066–1.98	0.068–2.04
Linear regression ^a^	y = 146,569x + 13,844	y = 91,237x + 9171	y = 83,358x + 6721	y = 222,660x + 26,077	y = 134,442x + 18,961
Correlation coefficient (r)	0.9957	0.9936	0.9929	0.9952	0.9936
Matrix-matched linear regression	y = 152,419x + 1710	y = 108,205x − 283	y = 76,980x + 6402	y = 257,580x + 18,065	y = 148,926x + 11,291
CF ^b^	0.96	0.84	1.08	0.86	0.90
Repeatability (%RSD)	3.3–6.6	2.7–5.4	3.7–6.3	2.0–7.3	3.9–6.1
Intermediate precision (%RSD)	<4.4	<7.2	<6.4	<6.9	<9.8
LOD (μg mL^−1^) ^c^	0.007	0.006	0.009	0.003	0.006
LOQ (μg mL^−1^) ^d^	0.02	0.02	0.03	0.01	0.02

^a^ Y: peak area, x: concentration (μg mL^−1^). ^b^ CF: correction factor. ^c^ Based on *S*/*N* = 3. ^d^ Based on *S*/*N* = 10.

**Table 3:** Accuracy data of the proposed HS-GC-ECD method for the quantitation of PGIs.

	Accuracy (%RSD)				
Added Concentration ^1^ (%)	nPrBr	isoPrBr	nBuBr	isoBuBr	secBuBr
5% (LOQ)	106.7 (3.3)	83.0 (2.7)	118.2 (3.7)	102.5 (3.8)	107.1 (3.9)
50%	111.2 (2.7)	90.9 (2.3)	116.8 (1.5)	101.6 (1.9)	109.9 (1.5)
100%	99.5 (2.2)	86.6 (5.4)	113.0 (2.9)	90.4 (6.1)	95.0 (5.4)
150%	88.7 (3.8)	86.7 (1.8)	100.5 (5.2)	81.5 (3.4)	85.2 (4.1)

^1^ Relative to the specification limit (1.3 μg mL^−1^) for each analyte.

## Data Availability

The data are available on request due to restrictions.

## Supplementary Materials

The following supporting information can be downloaded at: https://www.mdpi.com/article/10.3390/ph17040422/s1, Figure S1: Normal probability and the residuals vs. predicted plots for the peak area of A) nPrBr and B) isoPrBr. Figure S2: Normal probability and the residuals vs. predicted plots for the peak area of (A) nPrBr, (B) isoPrBr and (C) secBuBr. Table S1: Minimum-run (resolution IV) screening design for factor screening. Table S2: CCD experimental design and the obtained responses for each experiment. Table S3: ANOVA table for the peak area of nPrBr. Table S4: ANOVA table for the peak area of isoPrBr. Table S5: ANOVA table for the peak area of nBuBr. Table S6: ANOVA table for the peak area of isoBuBr. Table S7: ANOVA table for the peak area of secBuBr.
