# Supplementary material for: Analyzing Alkyl Bromide Genotoxic Impurities in Febuxostat Based on Static Headspace Sampling and GC-ECD
Source: Pharmaceuticals (Basel). 2024 Mar 26;17(4):422. doi: 10.3390/ph17040422 (PMC11053595; doi:10.3390/ph17040422)
Supplement: Supplementary file 1 [file pharmaceuticals-17-00422-s001.zip › pharmaceuticals-2897596-supplementary.pdf]

Supplementary material

Analyzing alkyl bromide genotoxic impurities in Febuxostat based on static headspace sampling and GC-ECD

Alexandros Kavrentzos [1,2], Elli Vastardi [2], Evangelos Karavas [2], Paraskevas D. Tzanavaras [3] and Constantinos K. Zacharis [1,*]

[1] *Laboratory of Pharmaceutical Analysis, Department of Pharmacy, Aristotle University of Thessaloniki, 54124 Thessaloniki, Greece; akavrentzos@pharmathen.com*

[2] *Pharmathen S.A. Pharmaceutical Industry, Dervenakion Str 6. Pallini Attikis, 15351 Athens, Greece; evastardi@pharmathen.com (E.V.); ekaravas@pharmathen.com (E.K.)*

[3] *Laboratory of Analytical Chemistry, Department of Chemistry, Aristotle University of Thessaloniki, 54124 Thessaloniki, Greece; ptzanava@chem.auth.gr*

***** *Correspondence: czacharis@pharm.auth.gr; Tel.: +30-2310997663*

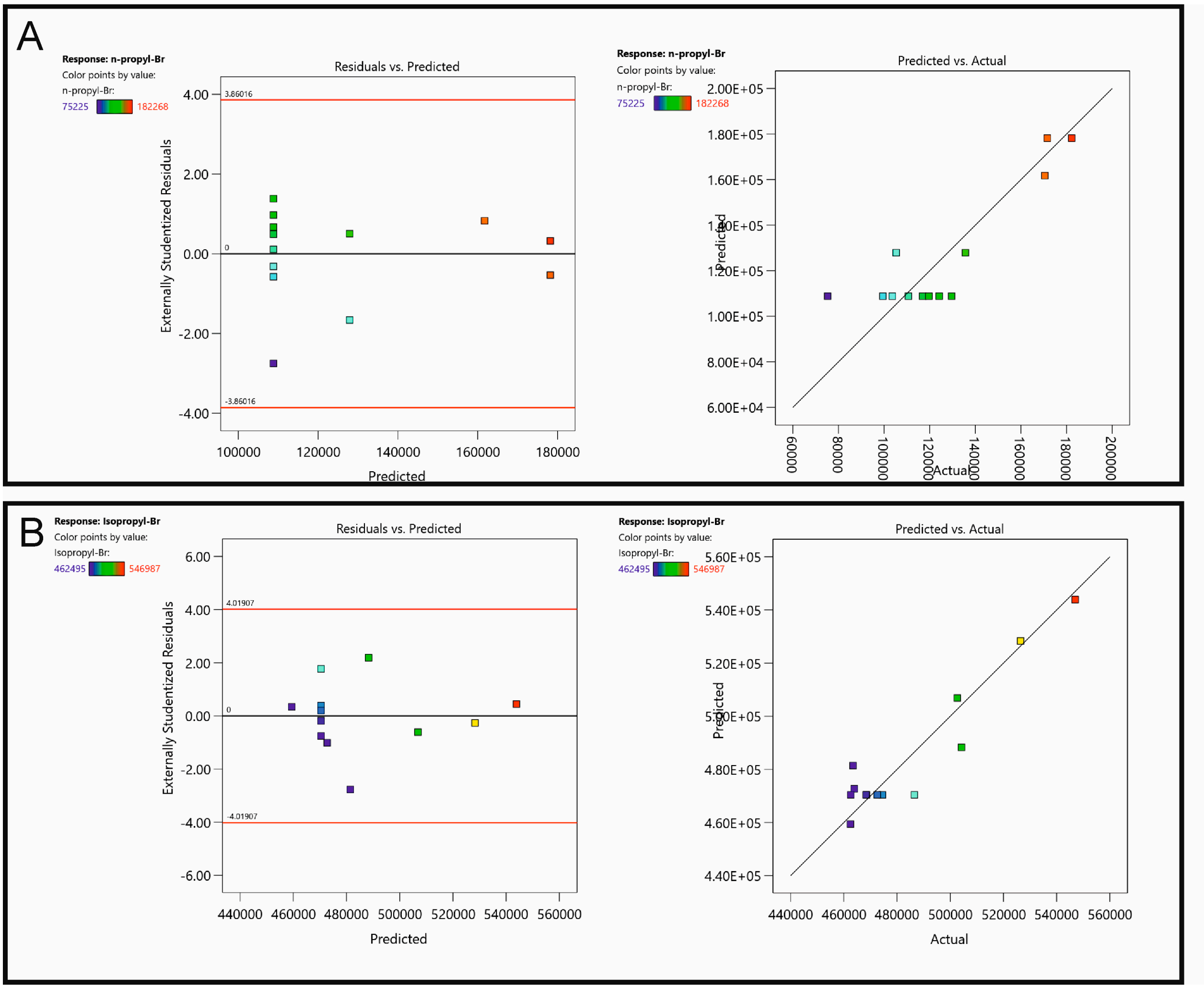

Figure S1. Normal probability and the residuals vs predicted plots for the peak area of A) nPrBr, B) isoPrBr.

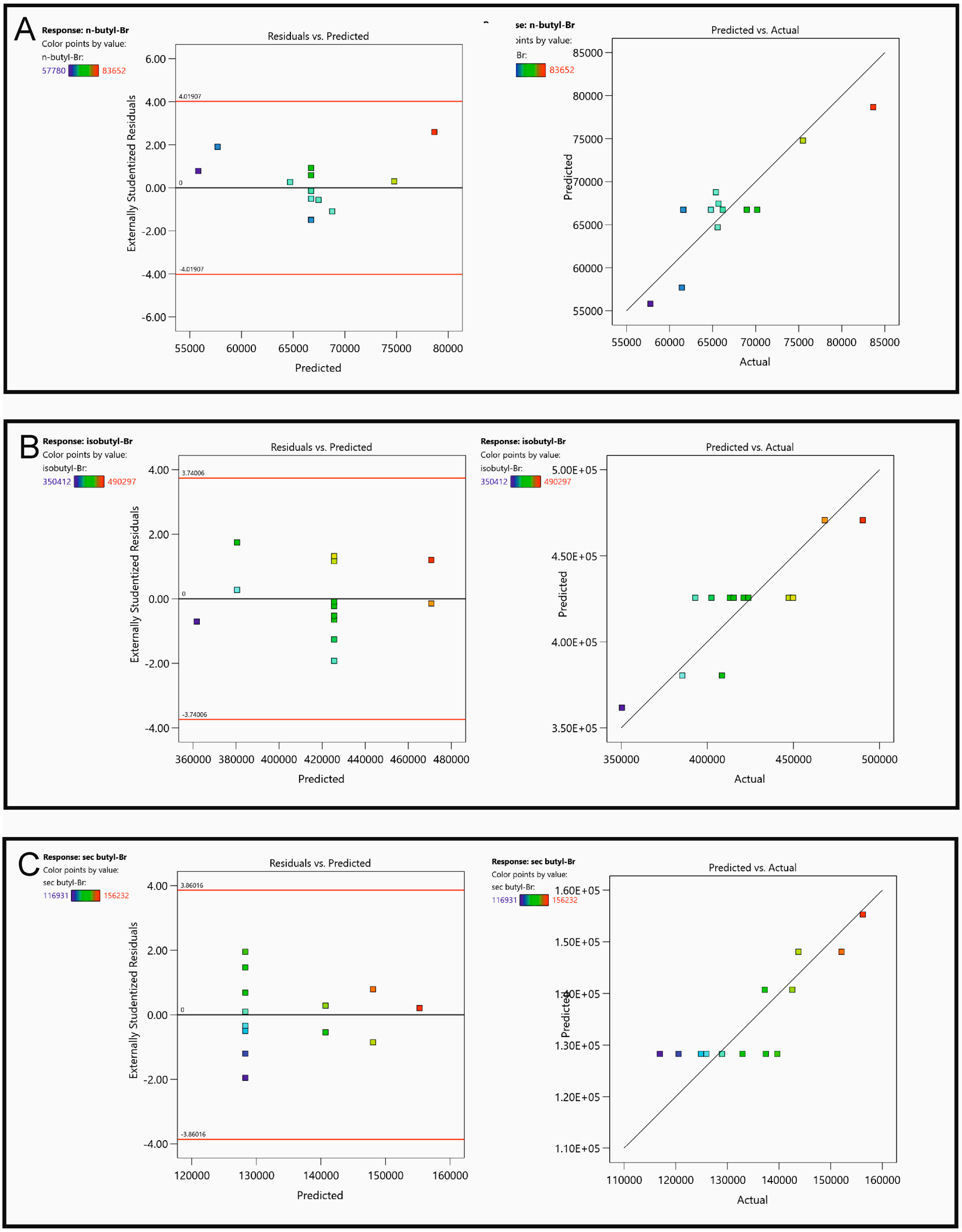

Figure S2. Normal probability and the residuals vs predicted plots for the peak area of A) nPrBr, B) isoPrBr and C) secBuBr.

Table S1. Minimum-run (resolution IV) screening design for factor screening.

Run No	Incubation temperature (°C) (Factor A)	Extraction time (min) (Factor B)	Agitation speed (rpm) (Factor C)	NaCl amount concentration (% m/v) (Factor D)	Sample volume (mL) (Factor E)	Peak area (nPrBr)	Peak area (isoPrBr)	Peak area (nBuBr)	Peak Area (isoBuBr)	Peak area (secBuBr)
1	80	30	250	10	1	113182	11488	62154	289783	120042
2	120	5	250	0	1	161203	142697	88770	384221	172060
3	120	5	750	0	5	166281	146836	94342	435652	183611
4	80	5	750	10	1	115320	109911	61160	265275	120983
5	120	30	250	10	5	37917	36845	27278	412577	54361
6	80	5	750	0	1	117758	112632	61804	268241	121371
7	120	5	750	10	1	166866	150820	99221	495110	192652
8	120	30	750	0	1	19376	24991	15240	399769	39786
9	100	17.5	500	5	3	120950	120705	68085	369021	139780
10	80	30	250	0	5	107558	109011	56499	270993	115837
11	100	17.5	500	5	3	120970	120725	68105	369041	139800
12	80	5	250	10	5	107828	106777	55108	240455	110301
13	80	30	750	10	5	115741	117861	60631	319257	110143
14	100	17.5	500	5	3	120920	120675	68055	368991	139750
15	120	30	250	0	5	52635	65772	36085	453137	76226

Table S2. CCD experimental design and the obtained responses for each experiment.

Run No	Incubation temperature (°C) (Factor A)	Extraction time (min) (Factor B)	Peak area (nPrBr)	Peak area (isoPrBr)	Peak area (nBuBr)	Peak Area (isoBuBr)	Peak area (secBuBr)
1	120	30	105307	463919	57780	408504	137252
2	100	17.5	103587	474526	68970	449766	120569
3	100	17.5	110667	472598	64796	423845	137453
4	100	17.5	99442	486521	61588	402364	128981
5	100	35.2	170458	502657	65680	350412	156232
6	100	17.5	75225	468574	66179	413078	124896
7	128.3	17.5	119667	462495	65588	447290	139669
8	100	17.5	124163	462587	61619	392994	116931
9	80	5	171573	546987	61425	490297	152109
10	71.7	17.5	116854	463469	65383	421050	125946
11	100	17.5	129611	468372	70171	415289	132944
12	120	5	182268	526434	75486	468170	143759
13	80	30	135647	504258	83652	385474	142569

Table S3. ANOVA table for the peak area of nPrBr.

**Analysis of variance table**						
**Source**	**Sum of squares**	***df*[1]**	**Mean square**	***F*-value**	***p*-value (Prob>*F*)**	
**Model**	9.094E+09	2	4.547E+09	16.21	0.0007	significant
B-Extraction Time	3.123E+09	1	3.123E+09	11.14	0.0075	
B^2	8.654E+09	1	8.654E+09	30.86	0.0002	
**Residual**	2.805E+09	10	2.805E+08			
Lack of Fit	9.070E+08	5	1.814E+08	0.4780	0.7815	not significant
Pure Error	1.898E+09	5	3.795E+08			
**Cor Total**	1.190E+10	12				

[1] degree of freedom

Table S4. ANOVA table for the peak area of isoPrBr.

**Analysis of variance table**						
**Source**	**Sum of squares**	***df*[1]**	**Mean square**	***F*-value**	***p*-value (Prob>*F*)**	
**Model**	7.925E+09	3	2.642E+09	22.75	0.0002	significant
A-Incubation temp	4.847E+08	1	4.847E+08	4.17	0.0714	
B-Extraction Time	3.818E+09	1	3.818E+09	32.88	0.0003	
B^2	6.343E+09	1	6.343E+09	54.62	< 0.0001	
**Residual**	1.045E+09	9	1.161E+08			
Lack of Fit	7.144E+08	4	1.786E+08	2.70	0.1527	not significant
Pure Error	3.309E+08	5	6.617E+07			
**Cor Total**	8.971E+09	12				

[1] degree of freedom

Table S5. ANOVA table for the peak area of nBuBr.

**Analysis of variance table**						
**Source**	**Sum of squares**	***df*[1]**	**Mean square**	***F*-value**	***p*-value (Prob>*F*)**	
**Model**	4.167E+08	3	1.389E+08	9.46	0.0038	significant
A-Incubation temp	1.659E+07	1	1.659E+07	1.13	0.3155	
B-Extraction Time	1.485E+06	1	1.485E+06	0.1011	0.7578	
AB	3.987E+08	1	3.987E+08	27.15	0.0006	
**Residual**	1.322E+08	9	1.468E+07			
Lack of Fit	6.700E+07	4	1.675E+07	1.29	0.3871	not significant
Pure Error	6.516E+07	5	1.303E+07			
**Cor Total**	5.489E+08	12				

[1] degree of freedom

Table S6. ANOVA table for the peak area of isoBuBr.

**Analysis of variance table**						
**Source**	**Sum of squares**	***df***[1]	**Mean square**	***F*-value**	***p*-value (Prob>*F*)**	
**Model**	1.191E+10	1	1.191E+10	30.64	0.0002	significant
B-Extraction Time	1.191E+10	1	1.191E+10	30.64	0.0002	
**Residual**	4.274E+09	11	3.885E+08			
Lack of Fit	2.348E+09	6	3.914E+08	1.02	0.5034	not significant
Pure Error	1.926E+09	5	3.851E+08			
**Cor Total**	1.618E+10	12				

[1] degree of freedom

Table S7. ANOVA table for the peak area of secBuBr.

**Analysis of variance table**						
**Source**	**Sum of squares**	***df***[1]	**Mean square**	***F*-value**	***p*-value (Prob>*F*)**	
**Model**	1.176E+09	2	5.879E+08	11.94	0.0022	significant
B-Extraction Time	6.681E+07	1	6.681E+07	1.36	0.2711	
B^2	1.145E+09	1	1.145E+09	23.24	0.0007	
**Residual**	4.924E+08	10	4.924E+07			
Lack of Fit	1.968E+08	5	3.935E+07	0.6654	0.6671	not significant
Pure Error	2.957E+08	5	5.914E+07			
**Cor Total**	1.668E+09	12				

[1] degree of freedom
